# Clinical Safety and Immunogenicity of Tumor-Targeted, Plant-Made Id-KLH Conjugate Vaccines for Follicular Lymphoma

**DOI:** 10.1155/2015/648143

**Published:** 2015-09-06

**Authors:** Daniel Tusé, Nora Ku, Maurizio Bendandi, Carlos Becerra, Robert Collins, Nyla Langford, Susana Inogés Sancho, Ascensión López-Díaz de Cerio, Fernando Pastor, Romy Kandzia, Frank Thieme, Franziska Jarczowski, Dieter Krause, Julian K.-C. Ma, Shan Pandya, Victor Klimyuk, Yuri Gleba, John E. Butler-Ransohoff

**Affiliations:** ^1^DT/Consulting Group, 2695 13th Street, Sacramento, CA 95818, USA; ^2^DAVA Oncology LP, Two Lincoln Center, 5420 LBJ Freeway, Suite 410, Dallas, TX 75240, USA; ^3^Ross University School of Medicine, P.O. Box 266, Portsmouth, Dominica; ^4^Baylor University Medical Center, C. A. Sammons Cancer Center, 3535 Worth Street, Dallas, TX 75246, USA; ^5^University of Texas, Southwestern Medical Center, 5323 Harry Hines Boulevard, Dallas, TX 75390, USA; ^6^Clínica Universidad de Navarra, Avenida Pío XII 36, 31008 Pamplona, Spain; ^7^CIMA, Universidad de Navarra, Avenida Pío XII 55, 31008 Pamplona, Spain; ^8^Icon Genetics GmbH, Weinbergweg 22, 06120 Halle, Germany; ^9^St. George's Hospital Medical School, Cranmer Terrace, London SW17 0RE, UK; ^10^Bayer Pharma AG, Gebäude 402, Raum 106, 42113 Wuppertal, Germany

## Abstract

We report the first evaluation of plant-made conjugate vaccines for targeted treatment of B-cell follicular lymphoma (FL) in a Phase I safety and immunogenicity clinical study. Each recombinant personalized immunogen consisted of a tumor-derived, plant-produced idiotypic antibody (Ab) hybrid comprising the hypervariable regions of the tumor-associated light and heavy Ab chains, genetically grafted onto a common human IgG1 scaffold. Each immunogen was produced in *Nicotiana benthamiana* plants using twin magnICON vectors expressing the light and heavy chains of the idiotypic Ab. Each purified Ab was chemically linked to the carrier protein keyhole limpet hemocyanin (KLH) to form a conjugate vaccine. The vaccines were administered to FL patients over a series of ≥6 subcutaneous injections in conjunction with the adjuvant Leukine (GM-CSF). The 27 patients enrolled in the study had previously received non-anti-CD20 cytoreductive therapy followed by ≥4 months of immune recovery prior to first vaccination. Of 11 patients who became evaluable at study conclusion, 82% (9/11) displayed a vaccine-induced, idiotype-specific cellular and/or humoral immune response. No patients showed serious adverse events (SAE) related to vaccination. The fully scalable plant-based manufacturing process yields safe and immunogenic personalized FL vaccines that can be produced within weeks of obtaining patient biopsies.

## 1. Introduction

Non-Hodgkin's lymphoma (NHL) is the sixth most common malignancy occurring in adults in the United States with a doubling of incidence since the 1970s [1]; in 2014, more than 70,000 new cases of NHL were diagnosed in the United States alone [2]. The worldwide incidence of NHL is estimated to be 6.1 per 100,000 in males and 4.0 per 100,000 in females with a mortality rate of 3.5 and 2.3 per 100,000 in males and females, respectively [1]. In the West, over 90% of NHL is comprised of B-cell lymphomas and the most common indolent B-cell lymphoma is follicular lymphoma (FL), which comprises approximately 22% of all B-cell lymphomas [3]. FL is an indolent yet incurable malignancy [4]. The typical clinical course of FL often spans over eight to twelve years during which time multiple lines of therapy can induce remission. Although successfully treated with chemotherapy given with or without rituximab, recurrence is common with each remission being progressively shorter in duration. The use of prolonged administration of rituximab improves event-free survival but not overall survival, and retreatment with rituximab at progression yields the same benefit as use of rituximab as maintenance therapy [5]. Hence, sustaining remission to improve overall survival has been difficult to achieve with available therapies. Strategies to improve the outcome of patients with FL are needed.

Follicular NHL is a clonal B-cell malignancy that expresses a unique idiotype “Id”: the antigen-binding site (variable region in light and heavy chains) in the antibody produced by the B-cell clone. The idiotype of a particular B-cell lymphoma has no known ligand but rather represents a tumor-specific antigen and, as such, presents a plausible target for clinical lymphoma treatment [6]. Taking advantage of this molecular targeting feature, experimental therapeutic vaccines against B-cell NHL have been designed to induce idiotype-specific immune responses to control the malignant clone specifically, without impact on the nonmalignant B-cell repertoire. Such tumor-targeted therapeutic vaccines have been produced using a number of different technologies, including human-mouse heteromyelomas [6–9], baculovirus-insect cell culture [10], and transient expression of the idiotype in green plants [11, 12], including our own prior work with agroinfection [13]. The idiotypic vaccines produced through these various platforms have been extensively studied in the clinic for more than 25 years, and, as reported in the above-referenced human and animal studies, proven to be safe and well tolerated [14].

Regardless of production platform, an individualized, custom-made idiotype vaccine for targeted therapy must be produced for each patient [15]. Therefore, any manufacturing method aimed at commercial implementation must be at a minimum (1) flexible, to express a multitude of individual patient-derived idiotypes; (2) robust, to accommodate heterogeneous physicochemical properties of the immunogen; (3) high-yielding, to provide minimum workable expression levels and recovery efficiencies; (4) rapid, to enable provision of vaccines to clinical centers quickly; (5) cost-effective, to make vaccination cost-competitive with current standards of care; and (6) quality compliant, to enable licensure of the vaccine product in multiple regulatory jurisdictions.

It may seem counterintuitive to consider the use of whole green plants as a viable platform relative to cell culture-based idiotype production systems, especially when considering the criteria of speed and the flexibility to manufacture a large number of small amounts of individualized proteins. However, in prior work some of us had shown that transient viral vectors could produce idiotype NHL immunogens in green plants rapidly and that the resultant vaccines met clinical safety and immunogenicity criteria [11, 12, 16]. The landmark study reported by McCormick et al. [12] represented the first plant-made vaccines to be parenterally administered to human subjects in a clinical trial under FDA IND. Notwithstanding the scientific significance of that study on plant-made NHL vaccines, the viral vectors used to manufacture the vaccines produced single-chain antibody fragments (scFv) representing the idiotype. Expression yields were highly variable [11, 13]; furthermore, the lack of a common molecular “handle” made standardized purification of each individual protein impossible and hence the system presented challenges in scalability, efficiency, and costs.

In the present study we demonstrate for the first time that a complex conjugate vaccine for targeted FL treatment can be produced in plants quickly using a fully scalable platform and in compliance with cGMP guidelines. We further demonstrate that such vaccines are safe and well tolerated and can induce tumor idiotype-specific humoral and cellular immune responses in a clinical study with FL patients. This is the first report of successful plant-based production of whole-antibody idiotype immunogens and their performance when administered to patients in a clinical study.

Vaccines for the present clinical study were produced using Icon Genetics' magnICON technology, a plant-based protein expression system that allows rapid and high-yield expression of exogenous proteins but does not result in genetic transformation of the plant; the advantages of magnICON vectors were reviewed by Gleba et al. [17]. Two plant viral expression vectors (one each for the light and heavy chain) encoding the tumor-derived idiotype immunoglobulin were used to transform* Agrobacterium tumefaciens*, which was in turn used to cotransfect the leaves of the plant host* Nicotiana benthamiana*, a close relative of tobacco (*N. tabacum*), using a vacuum infiltration step [17–19]. This transfection step induces the protein expression machinery of the host plant to express high levels of the tumor-derived immunoglobulin (idiotype). The transfected cells in the leaves of these plants are the equivalent to an individualized manufacturing cell line. Upon harvesting and extraction of the leaves and following standard immunoglobulin purification processes (i.e., protein A affinity chromatography, described in Bendandi et al. 2010 [13]), each tumor-derived immunoglobulin was chemically linked to the immunogenic carrier protein keyhole limpet hemocyanin (KLH). The manufacturing process was conducted in compliance with US FDA cGMP guidance. After quality release testing, the vaccines were shipped to the clinical trial sites for patient immunization in a Phase I safety and immunogenicity study.

This Phase I study's primary clinical goal was to demonstrate the safety of the recombinant autologous vaccines manufactured by the magnICON plant-expression technology (also known as “magnifection”) in patients who achieved a remission with a non-anti-CD20 based chemotherapy regimen. Secondary objectives were to evaluate idiotype-specific cellular and humoral immune responses to vaccination. In addition to assessing the immunogenicity of the vaccines, the main nonclinical goal of this study was to evaluate the performance and prospects of the magnifection process as a scalable, cost-efficient manufacturing platform for potential commercial implementation.

## 2. Materials and Methods

### 2.1. Vaccine Manufacturing

The manufacturing of idiotype vaccines in plants using magnifection technology was described previously [13] and is summarized below. Manufacturing was conducted by Icon Genetics GmbH (Halle/Saale, Germany) in compliance with FDA cGMP guidelines [20, 21].

The antigen used in these studies comprises an Id-containing IgG obtained by genetically fusing the variable region of the patient's tumor-specific Id with a generic constant domain of a human IgG1, regardless of the original tumor isotype (IgG or IgM; antigens of the IgA isotype were not included in this study). The variable light chain (*V*_*L*_) is fused to a generic kappa or lambda light-chain constant domain, depending on whether the patient's tumor-specific Ig contained a kappa or a lambda light chain. For the magnifection process, the variable regions of the Id were then subcloned in magnICON vectors (Icon Genetics, Halle, Germany) containing a plant signal peptide (rice or bean *α*-amylase) and a codon-optimized Ig constant region. The variable regions of the heavy and light chains were subcloned in both tobacco mosaic virus (TMV) and potato virus X (PVX) vectors, so that infiltration can be carried out with the heavy chain expressed in TMV and the light chain in PVX and vice versa. The TMV and PVX expression vectors were transformed into the industrial* Agrobacterium* strain ICF 320, a disarmed, auxotrophic derivative (Δ*cysK*_*a*_, Δ*cysK*_*b*_, and Δ*thiG*) of* Agrobacterium tumefaciens* strain C58, which was generated for this process. Both construct combinations were then delivered via* Agrobacterium* into* Nicotiana benthamiana* [18, 22] to test for the best possible combination of expression of the Id (data not shown). The above procedures resulted in successful expression of 21 patient-derived Igs of 21 attempted. Only one Ig could not be purified in sufficient quantity, so the final Ig manufacturing success rate was 20 Ig purified of 21 attempted.

A highly robust and reliable protocol for Ig purification based on protein A affinity capture was developed for molecules produced in this study. For Ig purification, 5-kg batches of green biomass were homogenized and acidified. Lowering the pH of the plant homogenate to <5.1, holding, and subsequently raising the pH to 8.5 followed by filtration enabled the removal of many highly abundant host cell proteins, for example, rubisco and larger debris. The resulting crude extract was suitable for subsequent chromatography. After protein A affinity capture, the Ig-containing eluate was further purified by membrane adsorption chromatography. This protocol enabled the reliable purification of Igs for vaccine manufacturing.

Each Id MAb was subjected to stringent quality control (QC) analyses, including appearance (visual), total protein (A280), purity (CGE and SEC-UV), identity (MW by SEC-LS; tryptic MALDI analysis of light and heavy chain fragments), endotoxin (harmonized method), residual DNA (threshold method), and sterility (harmonized method). MALDI MS analysis of purified IgG molecules cloned from all patients on study was used to calculate the differences between theoretical and determined MW caused by glycosylation. Glycosylation was further analyzed by LC-ESI-MS.

MAb immunogens that passed QC release criteria were subjected to KLH conjugation with glutaraldehyde, as described [13]. The conjugate vaccines were subjected to additional testing to meet specification. These vaccines comprised the drug products (final container) that were released for clinical administration.

### 2.2. Identity of Investigational Product

Genetic material required to produce each vaccine was produced from single-cell suspensions of lymphocytes taken from each patient's excised tissue and produced the same day as tissue procurement. These lymphocytes were sent under temperature-controlled conditions within 24 hours of collection and processing to the vaccine manufacturing facility at Icon Genetics in Germany. The resultant recombinant vaccine was supplied to the clinics in sealed, sterile glass vials.

Each vial of study drug contained 0.5 mg of idiotypic protein conjugated to 0.5 mg KLH in 1.0 mL clear phosphate-buffered saline with up to 0.3 mL overfill. Label information on each vial included batch number comprised of serial batch number and “UPIN” (a Unique Patient Identification Number), Study Number, contents labeled as “Recombinant Idiotypic Vaccine” and “New Drug” for investigational use, concentration of 1 mg/mL, date of manufacture, name of sponsor, and name of clinical research organization (CRO) overseeing study conduct. Study drug was stored frozen at the manufacturing facility until requested by the site. Vaccine vials were shipped with temperature recording at ≤−50°C on dry ice from the manufacturing facility to sponsor's CRO, which then transported the vials directly to study sites. At each site, study drug was kept at ≤−50°C until time of administration.

### 2.3. Study Objectives

#### 2.3.1. Primary

The primary objective of this clinical study was to evaluate the safety and tolerability to the magnICON produced Id vaccines administered with GM-CSF over a 6-cycle vaccination phase, wherein grade 3–5 adverse events are deemed to occur in <17% of patients and to be vaccine-related and unexpected as assessed by the FDA CBER Guidance for Industry for Toxicity Grading Scale in Preventive Vaccine Clinical Trials [23] and NCI-CTCAE version 4.02.

#### 2.3.2. Secondary

The secondary objectives were:

1. Assessment of humoral idiotype-specific immune response to vaccination defined as ≥40% of subjects developing a humoral immune response;
2. Assessment of cellular idiotype-specific immune response to vaccination defined as ≥50% of subjects developing a measurable cellular response;
3. Long-term safety/tolerability to the vaccines up to the conclusion of a 12-cycle vaccination phase, as determined by <17% of patients showing vaccine-related and unexpected grade 3–5 adverse events as assessed by the FDA CBER Guidance for Industry for Toxicity Grading Scale in Preventive Vaccine Clinical Trials and the NCI/CTCAE version 4.02.

### 2.4. Study Design and Rationale

This study was a single arm, repeated dose, nonrandomized Phase I trial evaluating the safety of the individualized recombinant idiotypic vaccines administered to patients in remission after treatment for relapsed follicular non-Hodgkin's lymphoma.

#### 2.4.1. Subject Enrollment, Inclusion, and Exclusion Criteria

Enrollment was open to patients with histologically proven follicular lymphoma relapsed after prior therapy or transformed follicular lymphoma relapsed after prior anthracycline therapy. Other key eligibility criteria at initial screening consisted of the following:

1. The tumor cells must express either an IgM or an IgG on their surface, but no IgA or lack of surface immunoglobulin.
2. Bone marrow involvement, known or unknown, is allowed.
3. Age > 18 years.
4. ECOG performance status of 0–2.
5. Life expectancy of at least 12 months.
6. Presence of at least a 2 × 2 cm in diameter single lymph node or equivalent volume of nodes accessible by physical examination for excision, for histological confirmation of diagnosis, and for manufacture of the vaccine.
7. No exposure to rituximab or anti-CD-20 directed therapy within 4 months prior to enrollment.

Additional exclusion criteria at initial screening or at time of initiation of vaccination included:

1. Development of intercurrent illness such that at the discretion of the investigator proceeding with chemotherapy or vaccination phases of the protocol would be detrimental;
2. Uncontrolled hypertension despite optimal treatment;
3. History of cardiac disease;
4. History of HIV (+), HBs antigen (+), or HCV (+);
5. Active clinically serious infections (>grade 2 NCI-CTC version 4.02).

#### 2.4.2. Study Design

**Figure 1:**
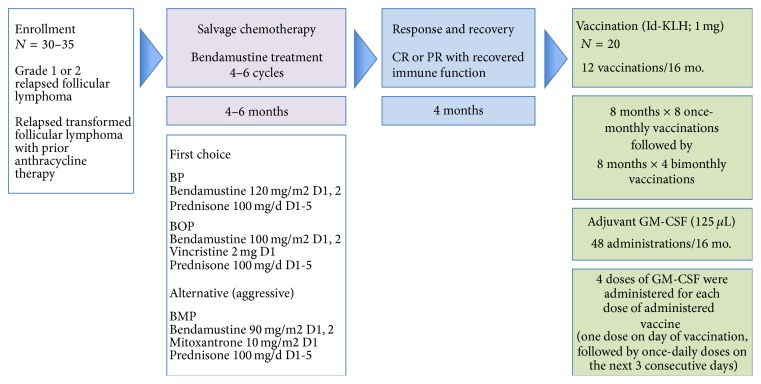
Study design summary: Phase I study of an autologous vaccine manufactured in plants by magnICON technology for the treatment of patients with relapsed or transformed follicular lymphoma.

The trial evaluated one-dose level of vaccine with no dose modification. The primary objective defined for the study was to evaluate safety and tolerability of the magnICON produced idiotype vaccine administered with GM-CSF over a ≥6-cycle vaccination phase when given to patients in complete remission (CR) or very-good PR (near-CR) following non-rituximab (non-anti-CD20) containing salvage chemotherapy for relapsed or transformed follicular lymphoma. Other objectives included immune response to the idiotype vaccine and long-term safety for patients who complete 12 doses of vaccine. The trial scheme is shown in Figure 1.

The trial design sought to identify and ultimately evaluate vaccine safety and immunogenicity in a relatively uniform group of patients. Thus, a single histologic subtype (follicular lymphoma), disease status (relapsed setting), and salvage therapy treatment (cytotoxic therapy with no anti-CD20 therapy) were required for participation. Avoidance of anti-CD20 therapy for at least 12 months prior to study drug exposure was incorporated into the study design. This was achieved by restricting eligibility to patients who had not received anti-CD20 therapy within 4 months of enrollment, using non-anti-CD20 containing salvage therapy for at least 4 months and incorporation of a 4-month observation phase prior to the start of study drug. This design was elected in an attempt to optimize each patient's ability to mount an immune response to study drug by avoiding the known long-lasting immunosuppressive effects of anti-CD20 therapy.

#### 2.4.3. Subject Withdrawal Criteria

Subjects could withdraw from the study at any time at their own request or be removed if, in the investigator's or sponsor's opinion, continuation in the study would be detrimental to the subject's well-being. Protocol specified reasons for study discontinuation were:

1. Patients not achieving a CR or PR after completion of salvage chemotherapy phase of this study;
2. Substantial noncompliance with the requirements of the study as defined by the coordinating investigator;
3. Patients with laboratory test results consistent with pregnancy. The pregnancy will be followed until delivery or resolution via the Pregnancy Monitoring Form. Pregnancy will be reported along the same time lines as a serious adverse event;
4. Use of illicit drugs or other substances that may, in the opinion of the investigator, have a reasonable chance of contributing to toxicity or otherwise skewing results;
5. Development of an intercurrent illness or situation which would, in the judgment of the investigator, interfere with the safety of the study subject on study therapy or affect assessments of clinical status and study endpoints to a significant degree;
6. The development of a second cancer;
7. Patient who is lost to follow-up;
8. Patient's death.

### 2.5. Study Sites

Patient recruitment and vaccine administration were performed at two sites in Dallas, Texas, United States, namely, The Simmons Cancer Center of the University of Texas Southwestern Medical Center and the Baylor Sammons Cancer Center. The conduct of this study at these sites adhered to ethical guidelines for research on human subjects in accordance with the Declaration of Helsinki (1964). The study was conducted with the understanding and the consent of each subject who elected to participate in the research (see next section). Each site's Institutional Review Board (Ethical Committee) reviewed and approved all aspects of the research prior to the enrollment and treatment of any subject.

### 2.6. Clinical Study Demographics

A summary of the demographic characteristics of patients enrolled on the trial is presented in Table 1. A majority of patients were male of age ≥50, as expected for the diagnosis of follicular lymphoma where the mean age of onset is 60 [1]. Although the trial sought and was available to persons of all ethnic groups, specific recruitment of minority groups was not pursued.

### 2.7. Subject Information and Consent

Prior to the commencement of any study specific procedures, including the study required lymph node excision, the PI or designee was responsible for proper informed consent and for obtaining a signed version of the ICF from each subject or legal representative.

The unique study design included at least an 8-month interval from study consent and enrollment prior to the start of investigational agent. Because of this intentional delay in consent until start of study drug, reconsent using the same consent form was required at a second screening step performed prior to start of study drug administration.

### 2.8. Clinical Study Conduct

This program was conducted in compliance with IND guidance under the jurisdiction of the Center for Biologics Evaluation and Research (CBER), US Food and Drug Administration (FDA), and was conducted in compliance with clinical trials registration guidelines. This study was registered with ClinicalTrials.gov in November 2009 with identifier NCT01022255. In addition, the clinical study was conducted in compliance with consolidated ICH guidelines for Good Clinical Practice [24] and all aspects of the clinical work were doubly monitored by each center's Institutional Review Board (IRB) and by an independent Clinical Research Organization (CRO) on behalf of the study's sponsor.

For operational purposes the trial was defined by the following phases.

#### 2.8.1. Screening Phase

This phase of the study included obtaining patient consent, performing screening tests, obtaining patient-specific tissue samples by outpatient surgical biopsy, and determining eligibility for the study based on screening test results and histologic information from tissue obtained at surgical biopsy. Adequate tissue was obtained at biopsy for autologous vaccine production.

#### 2.8.2. Chemotherapy Phase

As the effects of chemotherapy were not the focus of this study and the agents were FDA approved, this phase of treatment could be given by a study investigator or by a noninvestigator oncologist in communication with a study investigator. Because no investigational agents were administered and chemotherapy was given per standard dose and schedule, data collection was limited and safety reporting was not required for this phase of the trial.

After tumor excision, each subject fulfilling screening requirements received a salvage chemotherapy regimen with bendamustine (Treanda), in combination with other approved chemotherapeutic agents, to debulk the tumor and induce a measurable partial (PR) or complete remission (CR). Anti-CD20 therapy was not allowed. Bendamustine-based chemotherapy was selected because in disease refractory to rituximab and alkylator agents, bendamustine demonstrated an overall response rate of 70–90% [25–27] with complete response rates approaching 35%. For this trial the regimen of bendamustine, vincristine, and prednisone (BOP) or bendamustine and prednisone (BP) were defined as first choice options for salvage chemotherapy to be given only for 4–6 cycles, as prolonged chemotherapy was felt to compromise immunologic recovery. Patients were restaged after cycle 4 of chemotherapy and those not responding optimally to BP (or BOP) could withdraw from the study or be given 2 cycles of bendamustine, mitoxantrone, and prednisone (BMP) as specified by protocol to try to achieve the required CR/very good PR at the end of the salvage chemotherapy phase.

Patients who progressed, had stable disease, or had less than a very good PR as best response to 6 cycles of chemotherapy were taken off study.

#### 2.8.3. Observation Phase

After conclusion of the chemotherapy phase, the patient was then monitored for general health and immune recovery for 4 months. Patients were clinically reassessed with scans and laboratory analyses at the end of the observation phase. If disease was in stable CR/very good PR at this assessment, the patient was then reconsented and started on study drug.

#### 2.8.4. Vaccination Phase

During this last phase of the study, patients received the study drug given by monthly subcutaneous injection × 8, followed by bimonthly injection × 4, for a total maximum of 12 injections over 16 months.

### 2.9. Study Site Logistics

The sponsor of this study was Bayer Innovation GmbH, a subsidiary of Bayer AG, Leverkusen, Germany. Eligible patients were consented at two clinical study centers and excisional biopsies of each patient's tumor were obtained by a single study designated surgeon at a single study designated outpatient surgery center located in Dallas, Texas, United States. Within 2 hours of excision, tissue was reviewed for histology and isotype determination. If these qualifications did not meet study eligibility the patient was categorized as a screen failure. Otherwise aliquots of these tissue samples were shipped within 24 hours via air courier under temperature-controlled conditions to Icon Genetics' manufacturing facility in Halle, Germany, and to University of Navarra's clinical analytics facility in Pamplona, Spain. The vaccines were manufactured in Germany and shipped under temperature-controlled conditions to the CRO in Dallas, who after verifying sample identity and quality control documentation, transported the vaccines under temperature controlled conditions to the investigational pharmacy of the clinical centers for storage and subsequent administration to patients. During the study, patient samples of serum and plasma as well as peripheral blood mononuclear cells were obtained at each protocol designated sampling point and stored frozen. These samples were then batch-shipped under temperature-controlled conditions to the University of Navarra's Laboratory of Immunology, for vaccine immune response analysis.

### 2.10. Vaccine Administration

At the time of each scheduled administration, one vial labeled with the verified patient identifier (UPIN) was removed from the investigational pharmacy freezer and set aside at room temperature to thaw prior to injection. One mL of vaccine solution was drawn into a syringe and administered within 30 minutes of thawing at the selected subcutaneous injection site. The 1 mL dose contained 1 mg protein comprised of 0.5 mg Ig conjugated to 0.5 mg KLH. This Phase I trial did not attempt to determine an optimal dose; the 1 mg dose was in the range of 0.5 to 2.0 mg used in other trials with similar compositions [6, 8, 10, 12]. The study focused instead on determining safety, tolerability, and immunogenicity of the plant-produced product and its impurities.

As in prior FL vaccine studies, GM-CSF was added as an adjuvant to optimize immunologic responsiveness. GM-CSF at 125 *μ*L was administered at the injection site once on the day of vaccination and once daily for the next 3 days following vaccine administration. Thus, each subject received 4 doses of GM-CSF for every dose of idiotypic vaccine.

A planned maximum of 12 vaccinations was to be given over 16 months for patients who remained stable during the vaccination phase of the study. Vaccines were given once every 28 days ± 3 days for the first 8 doses then every 56 days ± 3 days for the last 4 doses. Study subjects could withdraw from the study at any time or were removed for recurrent disease requiring therapy that appeared during the vaccination phase.

### 2.11. Immune Responses to Vaccines

The immunological responses to the study drug were assessed by laboratory measurement of humoral and cellular immune responses from samples taken at baseline and monthly just prior to each vaccination, throughout the vaccination period. In particular, idiotype-specific humoral immune responses were assessed in both pre- and post-vaccine sera (via ELISA). Humoral immune responses were also assessed against the immunogenic carrier protein KLH, which is a component of these conjugate idiotypic vaccines, as an indicator of the patients' immune status since KLH is a strong immunogen that typically yields a positive response. Cell Mediated Immunity assays (CMI), such as ELISpot, flow cytometry, cellular proliferation, and/or multiplex immunological assays were performed as applicable as primary or confirmatory methods to assess the overall immune response, as well as specific recognition of each patient's idiotype and tumor-specific antigens. Peripheral blood mononuclear cells were collected from each patient and analyzed for immune status to help correlate responsiveness to vaccination. Positive and negative response criteria for each patient followed convention, as described by Inogés et al. 2006 [8].

A successful immunologic endpoint of this study was defined as (a) greater than 40% of subjects developing humoral immune responses after receipt of vaccine 6, or (b) greater than 50% of subjects developing cellular immune response after receipt of vaccine 6.

### 2.12. Immune Responses to Plant Glycans

All recombinant idiotypic antigens used in this study were glycoproteins and comprised IgG that contained oligomannosidic as well as plant-type glycans, the latter including vacuolar and secretory type structures (data not shown). Patients' pre- and post-immune sera were tested by ELISA for binding to plant glycosylated glycoproteins. Analyses were done in triplicate using vaccinated patient sera diluted 1 : 2000. Two antigens were used as controls, horseradish peroxidase (a glycosylated plant enzyme), and a plant-produced idiotype. For patient U001, the control idiotype was U011; for all other patients, the control idiotype was U001. Detection of serum binding was with alkaline phosphatase-labeled antisera. For ELISA plates coated with horseradish peroxidase, an anti-human IgG Fc antiserum was used. For ELISA plates coated with control idiotype U001, an anti-human kappa antiserum was used, and for control idiotype U011, an anti-human lambda antiserum was used. The presence of plant glycans on ELISA plates coated with horseradish peroxidase or control idiotypes was confirmed using a rabbit antiserum raised against* Phaseolus vulgaris* lectin that is known to bind to plant complex glycans. Detection was with anti-rabbit IgG antiserum labeled with alkaline phosphatase.

### 2.13. Statistical/Analytical Issues

The study population was too small to apply statistical analyses, as defined per protocol. Specific analytical issues (e.g., availability of lymphoid samples; minimum lymphocyte counts; success rate in vaccine production; reproducibility) are discussed in the following sections.

## 3. Results and Discussion

### 3.1. Manufacturability of Vaccines

Each personalized conjugate vaccine was manufactured and released within approximately 12 weeks of biopsy material reaching the manufacturing facility. This time reflects patient-specific sequence determination, expression of the idiotypic Ig (tumor-specific antigen) in plants and its purification, which required 2 weeks, and the time required for KLH conjugation of each antigen, purification, and quality control analyses for cGMP-compliant vaccine release.

The plant-based system used to manufacture the Ig for this study was capable of expressing all (21 of 21) idiotypic antigens scheduled for cloning. Of the 22 biopsies that were received at the manufacturing facility, one was found to code only for the Ig heavy chain and was rejected because it did not meet the idiotype specification; of the remainder, all 21 could be expressed* in planta*. Of those 21, only 1 could not be purified in sufficient quantity to develop a vaccine. In five instances total, patient biopsy material was difficult to obtain, contained insufficient cells from which to clone the idiotype, or was unavailable for other technical reasons and hence no vaccines were produced. These constituted “screen failures” and were not counted in the vaccine manufacturing tally (Table 4).

**Figure 2:**
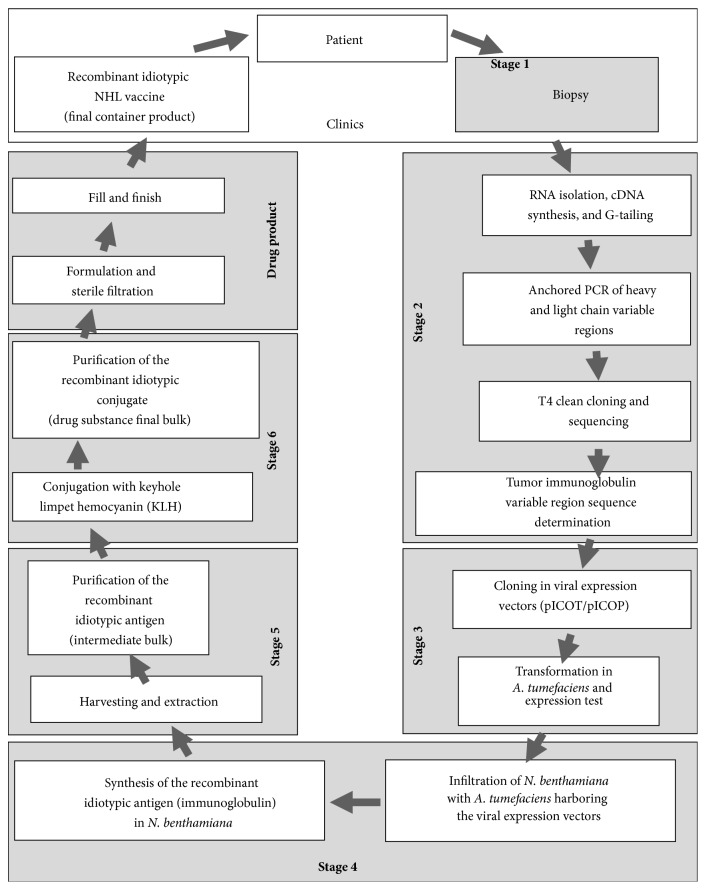
Individualized NHL vaccine manufacturing process overview.

An overview of the unit operations of the NHL vaccine manufacturing process employed is presented in Figure 2.

The speed, efficiency, and yield achieved with the magnICON expression system in producing patient-customized clinical materials for this study compared favorably with results we reported during developmental studies [13], in which we showed that all attempted vaccine antigens (22 of 22), including 20 human lymphoma-derived Ig and 2 murine lymphoma-derived Ig, were successfully produced. In the current study, 20 of the 21 Ig were successfully purified and released with cGMP compliance in sufficient yield to enable vaccine product manufacturing.

### 3.2. Patients Receiving Study Drug

The study initially planned to enroll approximately 30–35 patients to undergo chemotherapy (Figure 1) and to ultimately select 20 patients to receive vaccination, expecting that approximately 10 patients would drop from study due to disease progression, removal of consent, health complications not related to therapy, and other reasons. In actuality 27 candidates were screened, consented, and were enrolled. Five patients who initially met enrollment criteria failed laboratory screening tests specifically in the setting of node excision. Fourteen patients did not receive any study drug due to health reasons or progression prior to study drug administration. At the conclusion of the study, 135 vaccine administrations were given to 15 patients who had received at least one dose of vaccine, and 8 patients completed all 12 planned vaccinations, as summarized in Table 2. Eleven patients of the 15 patients receiving study drug received a minimum of 6 vaccinations and became evaluable for immune response to vaccination.

### 3.3. Safety and Tolerability

#### 3.3.1. Chemotherapy Phase

In this study, non-rituximab-based salvage chemotherapy was administered prior to initiation of vaccination. Adverse events and serious adverse events (AE/SAE) observed during the chemotherapy phase of the trial were not unexpected and were typical for those observed with the drugs used (data not shown). This trial was not designed to assess the efficacy of approved drugs for salvage therapy; nevertheless a valuable metric was derived with respect to immune depletion and its potential impact on immune response to vaccination. Most patients receiving vaccination were lymphopenic to various extents even after 4 months of recovery after receiving the last dose of chemotherapy (Table 4).

#### 3.3.2. Vaccination Phase

Adverse events were reported as defined by the 2007 CBER FDA grading scale for preventative vaccines. If toxicity was identified but not described in the CBER grading scale, the event was graded per the NCI CTCAE v4.02. Adverse events reported originally as a lower grade and subsequently worsening were captured twice, at the original lower grade and again at the worst grade. Adverse events which improved after initial reporting were captured only once at the original worst grade. Serious and nonserious adverse events were recorded during the vaccination phase of the trial for any patient receiving any dose of vaccine from 1 to 12. A total of 443 systemic and local nonlaboratory AE were reported regardless of attribution with 278 categorized as possibly, probably, or definitely related to vaccination.

These 278 nonlaboratory AE can be further categorized as 182 systemic events and 96 local injection reactions; only 21 total AE were grade 3 or 4 in severity (<7.5% for the entire study population). Grade 3 or 4 AE were seen in only 3 patients and only 21 reactions were considered related to study drug.

Table 3 is a summary of adverse events reported throughout the study.

Related grade 3 and 4 adverse events occurred as local injection site reactions, systemic symptoms, and musculoskeletal symptoms. Local injection site reactions related to study drug were reported if occurring within 7 days of study drug administration; related grade 3 reactions consisted of hardness, pain, redness, and swelling. The only grade 4 event definitely related to vaccination, but not unexpected, was an injection site reaction (injection site redness) occurring in one patient. Systemic symptoms related to study drug were all grade 3 and consisted of fatigue, non-cardiac chest pain, and generalized pruritus across 2 patients. One musculoskeletal grade 3 event, possibly related and not expected, was defined as musculoskeletal neck pain. No grade 3 or 4 events were reported as infections, gastrointestinal, or neurologic toxicities.

In this proof-of-concept trial adverse events categorized as related or possibly related to vaccine occurred in only 3 individual patients; thus, characterization of AE by gender, age, or race is not considered illustrative.

It is important to highlight that each vaccine dose was coadministered with 4 doses of the adjuvant GM-CSF. Therefore, a patient receiving the full course of 12 vaccines would also receive 48 injections of GM-CSF. The attribution of toxicities to the vaccine itself versus the GM-CSF administered concomitantly was difficult to separate and the adverse event profile gathered reflects toxicities for both.

In general, the adverse reactions to the vaccines were not unique from those known to be attributable to GM-CSF and can be easily compared to those toxicities summarized in the product monograph for intravenous Leukine when administered to enhance cytologic recovery post autologous and allogeneic stem cell transplant [28]. Side effects occurring in more than 30% of patients receiving GM-CSF include diarrhea, local reactions such as swelling, redness, and tenderness, and systemic reactions such as fatigue and weakness. Less common effects occurring in 10–29% of patients receiving GM-CSF have been reported to be flu-like symptoms (fever, generalized aches and pains, weakness, and fatigue) and edema of hands and feet. All of these same toxicities were reported for patients receiving the magnICON produced idiotype vaccine followed by GM-CSF. A total of five (5) SAE occurred during the vaccine phase of the study, none of which were attributed to vaccine administration.

Taking results obtained during this trial in their totality, we assert that this study met its primary and secondary safety objectives, namely, short-term and long-term safety and tolerability. The primary objective was to document safety and tolerability of the vaccines over a 6-cycle vaccination phase, defined by the incidence of vaccine-related, unexpected grade 3–5 adverse events occurring in <17% of patients receiving 6 vaccinations. In this study, 11 patients received a minimum of 6 vaccinations and became evaluable; thus, fewer than 2 patients (11 × 0.17 = 1.87 patients) were to experience vaccine-related, unexpected grade 3–5 events during the first 6 vaccinations for this vaccine to be considered safe. The primary endpoint of the trial was achieved; in the 11 patients receiving up to 6 vaccinations, grade 3–5 study drug-related and unexpected events occurred in only one patient.

This study also met its secondary objective for long-term safety/tolerability, as determined by the proportion of patients with toxicities as assessed by the NCI/CTCAE version 4.02 grade ≥3 to the magnICON-produced Id vaccine up to the conclusion of a 12-cycle vaccination phase (month 16). No (0) vaccine-related, unexpected grade 3–5 AE were reported in the 8 patients who completed the full course of 12 vaccinations throughout the entire trial.

### 3.4. Immune Responses 

#### 3.4.1. Overall Findings

While not designed to assess efficacy, this study evaluated immune responses to tumor antigen-specific vaccination with study drug. Serum samples from all 11 patients who received a minimum of 6 vaccinations were subjected to both humoral and cellular immune response analyses. Of the 11 patients evaluated, nine (9) responded to vaccination (82%). Three (3) of those patients (U001, U011, and T021) mounted both idiotype-specific humoral and cellular immune responses; this represents a 27% double-positive response rate to the vaccine. Nine (9) of 11 patients (82%; patients U001, T006, T010, U011, U016, U017, T021, T022, and U026) mounted an idiotype-specific cellular response. No (0 of 11) patients mounted only a humoral response without also mounting a cellular response. Two (2) of the 11 patients failed to respond to vaccination (18% non-responsive).

For comparative purposes in this research, we also developed hybridoma-produced idiotypes as counterparts to the plant-produced immunogens. These hybridoma-produced idiotypes were used to more specifically characterize patient sera for immune reactivity to the plant-produced idiotypes used in vaccination. Reactivity of patient sera to the immunogenic carrier protein KLH, which is conjugated to every idiotypic Ig and is part of the vaccine composition, was also assessed. Our observations from these comparisons are summarized for each patient evaluated.

The overall results obtained are summarized in Table 4. In the table, a successful immunologic response is marked as “+” and a negative response is marked as “−” following convention and criteria described by Inogés et al. 2006 [8] and others [12, 29].

#### 3.4.2. Interpretation of Immune Responses to Idiotypic Antigens

As expected, administration of personalized vaccines to patients with lymphoma produced a range of humoral and cellular responses. The results summarized in Table 4 are interpreted as follows for the 11 evaluable patients on study who successfully received a minimum of 6 vaccine administrations.

*(1) Strong Responders*. Three of the 11 evaluable subjects, namely, patients U001, U011, and T021, developed strong tumor idiotype-specific humoral and cellular responses (double positive) with two (U001 and U011) showing humoral responses against the carrier protein KLH, which is a component of all the plant-made conjugate vaccines. Patients U001 and U011 received the full course of vaccination (12 vaccines administered of 12 planned). The humoral response in both of these patients was positive not only to the plant-produced idiotype, but also to a corresponding hybridoma-derived idiotype developed only for comparative purposes. While the two idiotypes are identical, they are grafted onto different scaffolds: IgG for the plant-produced Ig, IgM for the hybridoma-produced Ig; the latter being the original, tumor-specific idiotypes derived from IgM-expressing tumor clones for both patients. 

Patients U001 and U011 also showed strong cellular responses to their respective idiotypes, with T-cell proliferation being positive at all pertinent time points when activated autologous tumor cells were used as stimulants. ELISpot confirmatory results were negative for U001 and positive for U011. Patient U001 had a normal or close-to-normal total lymphocyte count (except for low CD4+ T cells) both at screening and throughout most of the vaccination calendar. Patient U011 was lymphopenic (all T-cell subsets) throughout most of the vaccination calendar yet both humoral and cellular responses were detected.

Patient T021 received 9 vaccines of 11 scheduled, having missed vaccines 5 and 7 due to missed appointments and vaccine 12 due to progression. Patient T021's idiotype-specific humoral response was positive by ELISA but negative by flow cytometry, and similarly the patient's idiotype-specific cellular response was positive by T-cell proliferation but negative by ELISpot. Humoral response to KLH was also negative. This patient had a normal total lymphocyte count (except for low CD4+ T- and borderline CD19+ B-cells) both at screening and throughout most of the vaccination calendar yet both humoral and cellular idiotype-specific immune responses were detected, however, only by one of the two analytical methods employed. 

*(2) Intermediate Responders*. About one-half of subjects (6 of 11) in this study exhibited mixed responses to vaccination. For example, patients T006, T010, and U017 uniformly showed negative idiotype-specific humoral responses. Patients T006 and U017 received all 12 vaccines in the series, whereas patient T010 received 9 vaccinations before progressive disease caused removal from study. Patients T006 and U017 showed positive humoral responses to KLH, while T010's KLH response was negative. However, all three patients showed strong idiotype-specific cellular responses as assessed by T-cell proliferation as well as by confirmatory ELISpot. Patient U017's malignant tissue displayed both IgM-kappa and IgG-kappa isotypes and consequently two vaccines were produced. All three patients had normal total lymphocyte counts (except for low CD4+ T cells) both at screening and throughout most of the vaccination calendar.

Patients U016, T022, and U026 received a full course of 12 vaccines; none displayed a positive idiotype-specific humoral response to either the plant-made or hybridoma-made idiotype, but all showed a positive idiotype-specific cellular response as assessed by T-cell proliferation; responses were negative by ELISpot. Only sera from patients U016 and U026 had a positive response to KLH; T022's response was negative. Patients T022 had a normal total lymphocyte count but low CD4+ T- and CD19+ B-cells, while patients U016 and U026 were considerably lymphopenic (all T-cell subsets) throughout the calendar. 

*(3) Nonresponders*. A third group of subjects (2 of 11) comprised patients who did not respond to vaccination. Patient T003 had no measurable humoral or cellular idiotype-specific response throughout the vaccination calendar, in spite of having received the full course of 12 vaccines. Similarly, T008 showed no idiotype-specific humoral or cellular response after receiving 7 of 12 vaccines; the patient progressed after vaccination 4 but went on to receive 7 vaccinations before being removed from study and offered additional systemic therapy. Both patients did exhibit a humoral response to KLH albeit with a substantial delay. T003 was considerably lymphopenic throughout the calendar. Patient T008 was lymphopenic (except for the number of CD8+ T cells) with a particular depletion of helper T cells. No vaccine-induced immune response was ever detected and this fact seems consistent with a shortage of both B and T cells.

#### 3.4.3. Lack of Immune Responses against Plant Glycans

All idiotypic antigens contained plant-type glycans, including vacuolar type and secretory type complex structures. The reactivity of each patient's immune serum against plant-type glycans was determined by ELISA to further assess potential safety issues borne from plant-specific glycosylation of the antigens. This was seen as particularly poignant in a clinical study of vaccines comprising an immunogenic carrier protein (KLH) and coadministered with a potent cytokine adjuvant (GM-CSF), where immune reactivity against the complex is the desired goal of the treatment.

Table 5 shows the results of this analysis. In each case, antigen coating of the ELISA plates was confirmed by the control antisera. These assays were also used to confirm the absence of nonspecific cross-reactivity from the detecting antisera (see Section 2.12 for detail). Pre- and post-immune sera were analysed for the 11 evaluable patients on study. No evidence for an antibody response to plant glycans, either on the idiotypic vaccine or on horseradish peroxidase, was found in any patient. Some patients had preexisting low titres of glycan-binding antibodies, but the titre was unchanged between pre- and post-immunization sera.

## 4. Conclusions

We can conclude that the current tumor-targeted, plant-made vaccines manufactured with magnICON technology and administered in combination with the adjuvant GM-CSF are safe when given to adults with relapsed or transformed follicular lymphoma and that the vaccines induce tumor idiotype-specific immune responses. The percentage of patients who successfully mounted a documented vaccine-induced, idiotype-specific immune response (82% positive humoral and/or cellular) compares very favorably with the results of previously published clinical trials with FL idiotype vaccines produced through other manufacturing platforms (e.g., 80% immune response rate, hybridoma-produced, Inogés et al. 2006 [8]; 70% response, plant-produced scFv, McCormick et al. 2008 [12]; 70% response, hybridoma-produced, Inogés et al. 2009 [29]), and is in the range of other FL vaccines administered after non-rituximab-based chemotherapy, as reviewed by Park and Neelapu [30]. In our study, a successful immunologic endpoint was defined as (a) greater than 40% of subjects developing humoral immune responses after receipt of vaccine 6, or (b) greater than 50% of subjects developing cellular immune responses after receipt of vaccine 6. By these criteria, humoral responses were lower than expected but cellular responses exceeded expectations.

The overall immunogenicity to our current vaccine plus adjuvant combination might have been even higher had the patients been monitored for immune responsiveness (immune recovery after chemoreductive therapy) prior to initiation of the vaccination regimen. In relation to a similar study [8] in which 20% (5 of 25) of patients failed to respond to the administered vaccine, in the current study a comparable 18% (2 of 11) evaluated patients seemed to similarly fail, or at least fail to demonstrate, a vaccine-induced, idiotype-specific immune response as assessed by the complementary and independent functional assays performed. One explanation for why we may not have observed a higher rate of double positive responses (i.e., humoral + cellular) is that, in the cited study [8], which featured a hybridoma-produced idiotype, no patient in complete remission (CR) was vaccinated until evidence of B- and T-cell recovery had been demonstrated. In contrast, in the protocol of the current study, the recovery period was fixed at 4 months for practical reasons. In reality, most patients initiated vaccination between 4 and 5 months after conclusion of chemotherapy. Nevertheless, this is shorter than the recovery period adopted in prior studies (e.g., Inogés et al. 2006 [8]; McCormick et al. 2008 [12]; the latter with a 6-month minimum), and it could be expected that the current study had a greater proportion of patients who were not yet immunologically competent (due to insufficient recovery period) at the time of vaccination. These observations are semiquantitative because of the relatively small cohort sizes in all these studies.

It is noteworthy that most patients who failed to mount an immune response by the sixth vaccination were also unable to mount an immune response during subsequent vaccinations. The one exception was patient U017, who was negative for both humoral and cellular responses during most of the calendar until cellular responses were verified during administration of the last vaccines in the series. This observation suggests that a patient's immune status at the beginning of the vaccination calendar may be crucial to the potential development of a vaccine-induced immune response. An ad hoc analysis of minimal residual disease (MRD) versus treatment, as a secondary indicator of efficacy, was added late in the study but yielded no conclusive trends due to the small sample size available for evaluation (data not shown). There is likely room for improving the immunological efficacy of the vaccine via a modified composition, a new adjuvant, or both, plus improving the scheduling for administration to coincide with immune responsiveness. By adopting such changes the clinical benefit of this targeted therapy could be demonstrated in future studies.

In addition to immune responses to the vaccines, this safety study evaluated immune responses to plant-type glycans in the vaccine compositions. Prior studies on plant glycoforms had suggested that certain characteristic linkages in plant-derived complex glycans, particularly *β*(1,2)xylose and *α*(1,3)fucose, might be allergenic in humans [31–34]. In our study, a complete analysis of glycosylation was conducted for every plant-manufactured Ig. Extensive heterogeneity was found in the glycan content of the Ig of different patient samples, including oligomannosidic and plant-type complex glycans as well as truncations and mixtures thereof (data not shown). Analyses were also conducted to determine if there was any correlation of glycan content and/or pattern in the plant-made vaccines with either clinical safety or immunogenicity (no correlation was found).

As summarized in Table 5, no significant responses were found in any of the patients against the plant glycans associated with any of the idiotype vaccines. In fact, there were no antiglycan responses regardless of whether individuals were strong, intermediate, or weak responders to the vaccines. The results suggest that plant complex glycans are poor or not immunogenic in humans. The prior studies on plant glycan immunogenicity focused on known allergens, and it has been suggested that glycan conformation and the presence of other antigenic determinants may be key to development of immune responses [35]. Our findings are similar to those of McCormick et al. [12], who observed that immunogenicity of plant-produced NHL vaccines was due to the polypeptide component of the idiotype immunogens and that neither safety nor immunogenicity correlated with glycan content or structure. Our results therefore corroborate the growing body of evidence that the plant-type glycan content or structure in plant-produced immunogens is not,* per se*, a safety risk in vaccines administered in low doses by subcutaneous injection, even in the presence of a strong adjuvant.

Our results also validated the significant differentiating features of the magnICON plant-based expression technology used to manufacture the vaccines for this study, including its speed, versatility, and scalability, which are necessary prerequisites for implementation of any customized manufacturing process. The described process is reliable and robust; the total manufacturing time starting from biopsy to a conjugated vaccine is <12 weeks and the expression and purification of antigen required only 2 weeks. The methodology described lends itself to the rapid production of individualized proteins, such as Id NHL vaccine antigens, as well as the prototyping and production of other antigens whose seasonal or mutational variabilities, such as viral pathogens, favor a rapid and flexible manufacturing platform. Together with our earlier findings [18], this process also represents a broadly applicable, robust, scalable, and cost-effective platform for manufacturing monoclonal antibodies in plants, including novel and biosimilar biologics and therapeutic enzymes [36]. Most of the process steps can be automated [37], with the potential application of robotic high-throughput sample processing and cloning adding to the appeal of this flexible platform.

We conclude that customized idiotype vaccines produced by means of the magnICON plant-based expression technology are readily and economically manufacturable and are safe, well tolerated, and immunogenic according to the dose, route of administration, adjuvant, and schedule employed in the current study.

## Figures and Tables

**Table 1:** Demographic information of enrolled patients (*N* = 27).

Category	Number of subjects
Age group	
18–33	0
34–49	3
50–65	13
>65	11
Gender	
Male	18
Female	9
Race	
Caucasian	24
Asian	0
Hispanic	2
African American	1
Other	0

**Table 2:** Patient recruitment summary.

Planned	Screened/consented	Screen failure	Off study	Vaccinated	Completed study
30	27	5	14	15	8

**Table 3:** Summary of adverse events reported during the vaccination phase of the study.

	Maximum CBER/NCI CTC grade by patient				
	Total (related)	Grade 1	Grade 2	Grade 3	Grade 4
Local injection reactions	**109 **(96)	57	36	12	4
Systemic symptoms	**140** (85)	61	71	8	0
Infections	**11** (0)	2	9	0	0
Musculoskeletal	**127** (81)	58	62	7	0
Gastrointestinal	**42** (14)	30	11	1	0
Neurologic	**7** (2)	1	4	2	0
Other	**7** (0)	2	5	0	0
Total	**443 (278)**	**211**	**198**	**30 (20)**	**4 (**1**)**

Total number of adverse events (AE) by category reported throughout the study regardless of attribution. Numbers in parenthesis indicate AE that were possibly, probably, or definitely related to vaccination. Study safety objectives are defined by the frequency of vaccination-related grade 3–5 AE. No grade 5 AE occurred.

**Table 4:** Humoral and cellular responses to vaccination in the eleven evaluable FL patients on study.

Patient UPIN^1^	Tumor class	Number of vaccinations^2^	Immune status (lymphocyte number)	Humoral response^3^				Cellular response^8^	
				ELISA			Flow Cytometry^7^	T-cell proliferation	ELISpot
				KLH^4^	Plant Id^5^	Hybridoma Id^6^			
U001	IgM-lambda	12 of 12	Normal total lymphocyte count Low CD4+ T-cell count	+	+	+	+	+	−

T003	IgM-kappa	12 of 12	Lymphopenic (all subsets)	+	−	−	ND	−	−

T006	IgG-lambda	12 of 12	Normal total lymphocyte count Low CD4+ T-cell count	+	−	ND	ND	+	+

T008	IgM-kappa	7 of 12	Lymphopenic Normal CD8+ T-cell count	+	−	−	ND	−	−

T010	IgG-kappa	9 of 12	Normal total lymphocyte count Low CD4+ T-cell count	−	−	−	ND	+	+

U011	IgM-kappa	12 of 12	Lymphopenic (all T-cell subsets)	+	+	+	+	+	+

U016	IgM-lambda	12 of 12	Lymphopenic (all T-cell subsets)	+	−	−	ND	+	−

U017	IgM-kappa IgG-kappa	12 of 12	Normal total lymphocyte count Low CD4+ T-cell count	+	−	ND	ND	+	+

T021	IgG-lambda	9 of 11	Normal total lymphocyte count Low CD4+ T-cell count Borderline CD19+ B-cell count	−	+	−	−	+	−

T022	IgG-kappa	12 of 12	Normal total lymphocyte count Low CD4+ T-cell count Low CD19+ B-cell count	−	−	−	ND	+	−

U026	IgM-lambda	12 of 12	Lymphopenic (all subsets)	+	−	ND	ND	+	−

+ = Positive response; − = Negative response; ND = Not determined. 1 = Unique Patient Identification Number. 2 = Number of vaccines administered of the maximum 12 vaccines scheduled. 3 = Humoral responses were measured by ELISA and by flow cytometry. 4 = Specific response to KLH by ELISA was included to assess patients' overall immune responsiveness. 5 = Plant-made idiotype administered in the clinical study. 6 = Equivalent Id made in hybridoma to cross-check immune response to plant-made Id. 7 = Flow cytometry was used only as a confirmatory assay for humoral response when positive ELISA responses were measured. 8 = Cellular immune responses were measured by T-cell proliferation and by ELISpot.

**Table 5:** Lack of immune reactivity of patient sera to plant glycans in idiotypic vaccines.

Patient UPIN^1^	Predominant plant glycan type^2^	Vaccinated patient sera		Control^4^ glycosylated antibody pre	Control^4^ glycosylated antibody post
		Anti-HRP^3^	Anti-HRP^3^		
		Pre-immune	Post-immune		
U001	V: GnGnXF S: Gn(FA)XF; (FA)(FA)XF	0.076	0.068	0.071	0.065

T003	V: GnGnXF S: Gn(FA)XF; (FA)(FA)XF	0.081	0.103	0.084	0.080

T006	V: GnGnXF S: Gn(FA)XF	0.106	0.109	0.072	0.068

T008	V: GnGnXF	0.107	0.174	0.100	0.089

T010	V: MGnX S: GnAF/Gn(FA)	0.089	0.105	0.079	0.074

U011	V: GnGnXF S: Gn(FA)XF	0.078	0.069	0.156	0.130

U016	V: GnGnXF S: Gn(FA)XF	0.096	0.082	0.066	0.057

U017G	V: GnGnXF S: Gn(FA)XF	0.094	0.078	0.101	0.064

T021	V: GnGnXF	0.142	0.103	0.072	0.078

T022	V: GnGnXF	0.089	0.136	0.085	0.083

U026	V: GnGnXF S: GnA	0.067	0.069	0.062	0.066

Control anti-plant glycan antiserum (1 : 2000)		0.37		0.515	

1 = UPIN: Unique Patient Identification Number (sera obtained from patients vaccinated as shown in Table 4).

2 = Predominant plant glycan type. V, vacuolar, with major species indicated; S, secretory, with major species indicated. A = galactose; Gn = N-acetyl glucosamine; M = mannose; F = fucose; X = xylose.

3 = HRP, horseradish peroxidase, a plant enzyme containing complex plant-type glycans used as a control.

4 = Irrelevant plant-produced glycosylated human idiotypic Ig used as a control.
